# Non-Coding RNAs in Retinoblastoma

**DOI:** 10.3389/fgene.2019.01155

**Published:** 2019-11-14

**Authors:** Meropi Plousiou, Ivan Vannini

**Affiliations:** Biosciences Laboratory, Istituto Scientifico Romagnolo per lo Studio e la Cura dei Tumori (IRST) IRCCS, Meldola, Italy

**Keywords:** cancer, microRNAs, long ncRNAs, retinoblastoma, RB1

## Abstract

Retinoblastoma (Rb) is the most common ocular pediatric malignancy that arises from the retina and is caused by a mutation of the two alleles of the tumor suppressor gene, *RB1*. Although early detection provides the opportunity of controlling the primary tumor with effective therapies, metastatic activity is fatal. Non-coding RNAs (ncRNAs) have emerged as important modifiers of a plethora of biological mechanisms including those involved in cancer. They are classified into short and long ncRNAs according to their length. Deregulation of all these molecules has also been shown to play a critical role in Rb pathogenesis and progression. It is believed that ncRNAs can provide new insights into novel regulatory mechanisms associated with clinical pathological characteristics, facilitating the development of therapeutic alternatives for the treatment of Rb. In this review, we describe a variety of ncRNAs, which capable of regulating the most likely candidate genes involved in the tumorigenesis of Rb, could prove useful in analyzing different aspects of this cancer.

## Introduction

Retinoblastoma (Rb) is the most common primary ocular paediatric malignancy and the first to be identified as having hereditary features. Inactivation of the *RB1* gene is thought to be the main reason for the development of the tumor (Ma et al., 2014). The impact of Rb is substantial, with an incidence of nearly one in 15,000–20,000 births, resulting in 9,000 new cases every year (Kivelä, 2009; Thériault et al., 2014). Although it is possible to control the primary tumor with effective therapies, metastatic disease is still fatal. The survival rate is lower in under-developed countries because the disease is often diagnosed at later stages, and the mortality rate among children is 50%–70% (Jabbour et al., 2012).

Rb can be either non-hereditary or hereditary and can affect only one eye (unilateral) or both (bilateral). In particular, the bilateral forms of heritable Rb show a germline mutation of the *RB1* gene which is followed by a second somatic inactivation of the other allele. Conversely, somatic inactivation of both *RB1* alleles in retinal cells is responsible for non-heritable cases (Thériault et al., 2014). The *RB1* gene is considered to be a central regulator of the cell cycle mechanism and is inactivated in a wide range of cancers. Its tumor suppressor function is mainly known due to the inhibition of the E2F1 transcription factor, which in the absence of *RB1* pushes cells from G1 to S phase of the cell cycle. However, *RB1* appears to function in various ways, controlling more than four types of protein interactions and taking on the role of transcriptional co-factor and adaptor protein. For example, it represses *E2F1* gene target transcription through the recruitment of chromatin remodelling complexes including HDAC, DNMT1, HP1A, and SUV39H1 to promoters. With regard to non-E2F1 transcriptional factors such as MYOD, HIF1a, and SP1, *RB1* appears to act as a transcriptional co-factor (Burkhart and Sage, 2008).

Another important gene involved in Rb progression is the enhancer of zeste 2 polycomb repressive complex 2 subunit (*EZH2*), which was the first overexpressed epigenetic enzyme identified in Rb samples. *EZH2* is a histone methyltransferase expressed only in Rb and is essential for tumor development because of its capacity to silence tumor suppressors such as p19/p14ARF and p16/INK4a. Targeting EZH2 could be the basis for developing an epigenetic therapeutic approach in ocular oncology (Khan et al., 2015). However, new molecules that are involved in Rb are studied to improve the therapy.

Non-coding RNAs (ncRNAs) are transcripts that do not encode proteins. They are diffused in the human genome and abnormally dysregulated in cancer cells. Given that ncRNAs are often located in fragile sites (FRA), common breakpoint sites and in regions with loss of heterozygosity, they represent a new category of genes that participate in tumorigenesis (Calin et al., 2004; Calin et al., 2007). Some ncRNAs have an oncogenic function while others act as tumor suppressors (Sanchez Calle et al., 2018; Vannini et al., 2018a). ncRNAs are classified into two groups on the basis of the length of their sequence: short ncRNAs have a maximum length of 200 nucleotides and long ncRNAs (lncRNAs) are transcripts with sequences of over 200 nucleotides.

microRNAs (miRNAs), the most widely studied class of ncRNAs, are small molecules containing around 22 nucleotides. They regulate the expression of more than 60% of genes. miRNAs are included in the RNA-induced silencing complex (RISC), an miRNA effector machine. This complex binds the 3’ untranslated region or, less frequently, the 5’ untranslated region of mRNA target, determining the protein downregulation by mRNA degradation or translational repression. miRNAs also upregulate gene expression (Vasudevan et al., 2007). They are located in intergenic regions with independent promoters (Ambros et al., 2003) but can be transcribed by introns with the same promoter as that of the host gene (Lin et al., 2006).

lncRNAs are usually transcripts with 5’ terminal methylguanosine cap, frequently polyadenylated, and alternatively spliced (Spizzo et al., 2012; Ulitsky and Bartel, 2013). They have a thermodynamically stable secondary structure with bulges and hairpin loops (Mercer and Mattick, 2013) that enables them to interact with proteins, mRNAs, ncRNAs, and DNA. lncRNAs regulate gene expression at varied levels, from mRNA translation to cytoplasmatic and nuclear epigenetic processes such as miRNA sponging (Vannini et al., 2018b).

The present review summarizes the most important dysregulated ncRNAs in Rb, the interaction with some of their target molecules, and the mechanisms involved in tumor progression.

## miRNAs as Diagnostic and Prognostic Biomarkers in Rb

The formation and progression of numerous cancer types is frequently correlated with an altered miRNA expression profile.

A comparison by Beta et al. between miRNA profiles in primary Rb tissues and miRNAs detected in the serum of children with Rb revealed eight downregulated miRNAs (miR-216a, miR-217, let-7a, let-7i, let-7f, miR-9, miR-92a, miR-99b) and 25 upregulated (miR-103, miR-142-5b, miR-106b, miR-143, miR-148b, miR-17, miR-16, miR-183, miR-182, miR-19a, miR-18a, miR-29a, miR-29b, miR-29c, miR-20a, miR-30b, miR-30d, miR-34a, miR-494, miR-378, miR-513, miR-513-1, miR-513-2, miR-518c, miR-96) miRNAs. It would thus seem that these 33 RNA molecules are Rb-specific and could potentially influence tumorigenesis and tumor progression in the disease (Beta et al., 2013). Another study identified a group of 24 differentially expressed miRNAs (nine upregulated and 15 downregulated) in healthy retinal tissues and Rb tissues. Among these, 14 miRNAs including miR-20a, miR-373, miR-125b, let7a, let-7b, let-7c, miR-25, and miR-18a proved capable of distinguishing between Rb samples and healthy tissues, thus identifying potential biomarkers of Rb (Yang and Mei, 2015). Likewise, Liu et al. demonstrated that miR-320, let-7e, and miR-21 were disregulated in plasma of Rb patients and can thus be hypothesized as novel diagnostic biomarkers for the disease (Liu et al., 2014).

Despite the tumor heterogeneity of Rb, Castro-Magdonel et al. identified a common miRNA expression profile, highlighting miR-3613 as an interesting candidate for therapy. It was highly expressed in all of the examined samples and was also observed to have more than 36 tumor suppressor gene targets (Castro-Magdonel et al., 2017). The microenvironment was recently identified as one of the main factors influencing the background of many types of cancer. Hypoxia is considered as one of the first conditions of stress present in the tumor microenvironment. Interestingly, studies have shown that a hypoxic tumor microenvironment plays a crucial role in controlling treatment outcomes in Rb. It has, in fact, been associated with treatment failure given that it is capable of regulating various pathways including growth factor signalling, glycolysis, genetic instability, metastasis, and angiogenesis. Sudhakar et al. studied the expression of hypoxia-related proteins such as HIF-1A and survivin to understand whether hypoxia is present in Rb, observing that increased expression of these proteins induces resistance to cytotoxic therapy (Sudhakar et al., 2013). Various studies have shown that hypoxic conditions can modulate the expression of a group of miRNAs called hypoxia-regulated microRNAs (HRMs). More precisely, microarray analysis identified miR181b, miR30c-2, miR125a3p, miR497, and miR491-3p as the most important HRMs in Rb cells (Xu et al., 2011). By *in silico* and *in vitro* approaches, Venkatesan et al. identified two key miRNAs (miR486-3p, miR-532) that are downregulated in Rb. Their overexpression using mimic miRNA strategy on Rb cells led to apoptotic cell death (Venkatesan et al., 2015).

## miRNA Pathways in Rb

Although the critical role played by miRNAs in cancer has been demonstrated, further research is needed to clarify the link between cancer-related miRNAs and their target genes and to identify their correlation with multiple pathways associated with tumorigenesis. Through *in silico* and *in vitro* analysis of different cancer types it has been possible to identify and validate miRNAs that directly regulate RB1 gene (**Table 1**).

**Table 1:** List of validated/predicted microRNAs (miRNAs) that target the *RB1* gene.

miRNA	miRBase accession number/HGNC ID	Tumor type	Validated/Predicted miRNA (Reference/Database)
**miR-661**	**MI0003669/** **HGNC:32917**	**Lung cancer**	**Validated miRNA** (Liu F, et al., 2017)
**miR-215**	**MI0000291/HGNC:31592**	**Gastric cancer**	**Validated miRNA** (Chen et al., 2017)
**miR-221-3p**	**MI0000298/** **HGNC:31601**	**Pancreatic cancer**	**Validated miRNA** (Zhao et al., 2016)
**miR-132**	**MI0000449/HGNC:31516**	**Pancreatic cancer**	**Validated miRNA** (Park et al., 2011)
**miR-212**	**MI0000288/** **HGNC:31589**	**Pancreatic cancer**	**Validated miRNA** (Park et al., 2011)
**miR-675**	**MI0005416/HGNC:33351**	**Glioma**	**Validated miRNA** (Zheng et al., 2017)
**miR-3129-5p**	**MI0014146/** **HGNC:38217**	**–**	**Predicted miRNA** **(TargetScan Human)**
**miR-199a-3p**	**MI0000242/** **–**	**–**	**Predicted miRNA** **(TargetScan Human)**
**miR-199b-3p**	**MI0000282/** **HGNC:31573**	**–**	**Predicted miRNA** **(TargetScan Human)**

Lyu et al. observed low expression levels of miR-485 in Rb tissue and cell lines through reverse transcription-quantitative polymerase chain reaction (RT-qPCR). They also confirmed that miR-485 has a tumor suppressor function targeting Wnt family member 3A (Wnt3A) which activates the canonical Wnt signaling pathway, leading to decreased Rb proliferation, invasion, and migration (Lyu et al., 2019b).

Zhao et al. analyzed miR-361-3p expression levels by qRT-PCR in serum and tissue of Rb patients, in serum samples and normal retinal tissue from healthy controls, and in human Rb cell lines. The authors demonstrated that miR-361-3p was downregulated in Rb serum, Rb tissue and Rb cell lines compared with normal serum and normal retinal tissue. They also observed that miR-361-3p decreased Rb cell proliferation *via* targeting of GLI family zinc finger 1 and 3 (GLI 1/3) (Zhao and Cui, 2018).

Various studies have demonstrated that miR-183 is dysregulated in a great number of cancer types (Li et al., 2010; Lowery et al., 2010; Zhao et al., 2012). Interestingly, Wang et al. observed that miR-183 was downregulated in Rb cell lines and tissues with respect to healthy retinal tissues and that its forced overexpression inhibited the migration, proliferation and invasion capacity of Rb cells. Specifically, the mechanism underlying these results included low-density lipoprotein receptor-related protein (*LRP6*), a candidate target gene of miR-183 known to counteract the apoptotic effects of this miRNA. The authors showed that miR-183 directly targeted LRP6, downregulating its expression, and thus controlling the progression and development of Rb (Wang et al., 2014).

Another miRNA that has been shown to be involved in various cancer types, including Rb, is miR-204. Wu et al. reported that miR-204 is downregulated in Rb tissues and cell lines. The authors identified *cyclin D2* and matrix metalloproteinase *MMP-9* as its putative gene targets and focused on elucidating the mechanisms underlying this interaction (Wu et al., 2015).

MMP-9 is a protease that plays a strategic role in extracellular matrix (ECM) remodelling under normal conditions but also in the degradation of ECM by cancer cells during the metastasis process. Cyclin D2 is involved in the phosphorylation of RB1 and numerous studies have demonstrated that its expression levels are high in various cancers. It has emerged that cyclin D2, MMP-9, and miR-204 expression are inversely correlated and that miR-204 targets cyclin D2 and MMP-9, inhibiting tumor growth in Rb (Wu et al., 2015; Golabchi et al., 2017).

miR-29a expression is inversely correlated with cyclin D1 and matrix metalloproteinase (MMP-2) being part of signal transducer and an activator of transcription 3 (*STAT3*) downstream genes. Liu et al. demonstrated that miR-29a expression is very low in Rb (cells and tissues) and that STAT3 is a direct target of this miRNA (Liu et al., 2018).

Wang et al. discovered that miR-504 was decreased in Rb cell lines and tissue, reporting that miR-504 overexpression suppressed Rb cell proliferation and invasion. This suggests that miR-504 explicates its tumor suppressor function by directly targeting the astrocyte elevated gene 1 (AEG-1) known to be involved in the aggressiveness of Rb (Wang et al., 2019).

Guo et al. demonstrated that miR-98 suppressed Rb invasion, migration, and cell growth through the regulation of insulin-like growth factor1 receptor (IGF1R). IGF1R is a tyrosine kinase receptor that regulates IGF1-induced signaling events and plays an important role in cellular processes including differentiation, proliferation, and migration. The authors reported that miR-98 inhibited the k-Ras/Raf/MEK/ERK signaling pathway *via* targeting of IGF1R in RB. In fact, the restoration of IGF1R reverted the effects of miR-98 on Rb migration, invasion, and cell viability (Guo L, et al., 2019).

Sun et al. demonstrated that miR-492 was upregulated in Rb cell lines and tissue. Downregulating this miRNA, they confirmed its oncogene function that leads to a decrease in Rb proliferation and invasion. Using bioinformatic analysis and luciferase reporter assay, the authors observed that miR-492 targeted large tumor-suppressor kinase 2 (LATS 2), a serine/threonine protein kinase (Sun et al., 2019).

Another potential therapeutic axis was proposed by Zhang et al. for miR-125a-5p/TAZ and EGFR. The authors demonstrated that miR-125a-5p was markedly downregulated in Rb and that its overexpression inhibited cell proliferation. Transcriptional co-activator with PDZ-binding motif (*TAZ*) is a novel oncogene that promotes tumor progression and is one of the elements of the Hippo tumor suppressor pathway. TAZ is overexpressed in Rb and has been shown to stimulate tumor progression *via* the *EGFR* pathway. miR-125a-5p, in targeting TAZ, suppresses EGFR pathway and consequently Rb progression (Zhang et al., 2016). Whilst miR-125a-5p is downregulated in Rb, miR-125b is reported to be upregulated in both tissue and Rb cells. It has been shown to suppress cell apoptosis and promote cancer cell proliferation *via* interaction with one of its putative target genes, DNA damage regulated autophagy modulator 2 (*DRAM2*).

DRAM2 plays a crucial role in TP53-mediated apoptosis and induces cell autophagy. Studies have confirmed that DRAM2 is downregulated in various cancer types, indicating that it may be part of the tumor signalling process. *DRAM2* gene is linked to Rb because of its involvement in the renewal and recycling mechanism of photoreceptor cells located in the retina, an essential procedure for visual function preservation.

Bai et al. showed that miR-125b exerts its oncogene biological function by directly targeting and downregulating *DRAM2* gene expression, consequently suppressing cell apoptosis (Bai et al., 2016).

**Figure 1:**
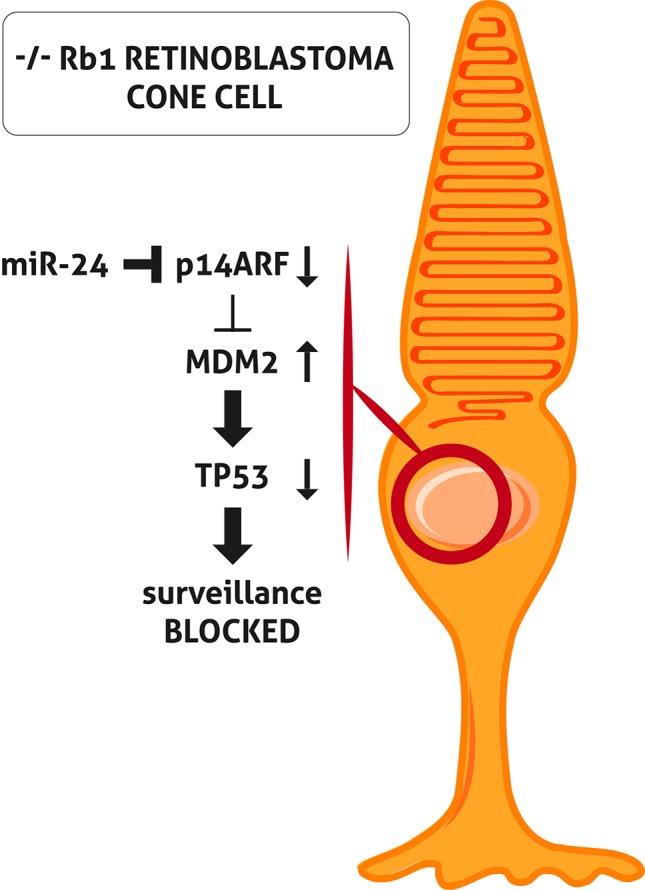
miR-24 blocks *TP53 s*urveillance through p14ARF in -/- RB1 retinoblastoma (Rb) cone cell. In Rb cone cell without *RB1*, miR-24 blocks p14ARF activation. Consequently, MDM2 level is increased and leads to *TP53* pathway block and inactivation of *TP53-* mediated surveillance.

The inactivation of both *TP53* and *RB1* pathways is an essential characteristic of tumorigenesis in the majority of cancer types. However, the classic *TP53* mutation does not occur in Rb. When *RB1* is inactivated, cells generally respond by activating *TP53* which induces cell cycle arrest and apoptosis. TP53 is regulated by nuclear-localized E3 ubiquitin ligase (MDM2), which targets TP53 for proteasomal degradation. In cells with a loss of *RB1*, E2F1 is activated and induces cyclin-dependent kinase inhibitor 2A (p14ARF), inhibiting MDM2 and leading to TP53 stabilization and TP53 target gene expression (Sherr, 2006). To et al. demonstrated that miR-24 represses p14ARF in Rb cells without *RB1* causing a block of activation of TP53 tumor surveillance (To et al., 2012) (**Figure 1**). This might explain the lack of mutation of the *TP53* pathway in Rb (Laurie et al., 2006; Xu et al., 2009; Ksander, 2010; Turhan, 2014).

miR-21 is reported to be one of the most dysregulated miRNAs in Rb. Shen et al. focused on this cancer-related miRNA and its potential gene targets in order to shed light on the complicated regulatory network. One of the identified gene targets is programmed cell death 4 (*PDCD4*), a well-known tumor suppressor gene that promotes cell apoptosis and inhibits cell migration and proliferation. Downregulation of PDCD4 expression has also been assessed in other cancer types including breast and lung cancer, hepatocellular carcinoma, and colorectal and squamous cell carcinoma. Shen et al. observed that miR-21 and PDCD4 were inversely correlated and that miR-21 directly targeted PDCD4 (Shen et al., 2014).

A regulator of Wnt signalling is Disheveled-Axin domain containing 1 (DIXDC1). Che et al. showed that DIXDC1 was significantly upregulated in Rb. They also observed that the expression of DIXDC1 and miR-186 was inversely correlated. miR-186 overexpression caused a downregulation of DIXDC1 and inhibited the proliferation and invasion in Rb cells (Che et al., 2018).

Wang et al. demonstrated that miR-138-5p has a tumor suppressor function, downregulating miR-138-5p and upregulating pyruvate dehydrogenase kinase 1 (PDK1) in Rb cells. PDK1 caused the phosphorylation of the pyruvate dehydrogenase enzyme. miR-138-5p overexpression led to a downregulation of PDK1 and consequently a decrease in migration, cell viability, invasion and induced apoptosis (Wang et al., 2017).

Montoya et al. elucidated the regulation of Rb proliferation by miR-31 and miR-200a, both of which were downregulated in Rb cell lines. Their induced overexpression by mimic transfection restricted the proliferative capacity of the Y-79 Rb cell line (Montoya et al., 2015).

miR-106b and its target gene Runt-related transcription factor 3 (Runx3) have also been identified as capable of modulating cancer progression. Runx3 is a tumor suppressor in Rb with an important role in mammalian development. Its loss is linked to a variety of cancers such as gastric cancer, glioblastoma, and bladder tumors. Yang et al. demonstrated that miR-106b binds to the Runx3 and downregulate its expression, which consequently leads to the development of the malignant phenotype. Inhibition of miR-106b and thus upregulation of Runx3 could represent be a possible therapy for Rb. (Yang et al., 2017).

Moreover, given the important role of the miR-17-92 cluster in the development of Rb, it is opportune to mention the advantage of the aptamer method, elucidated by Subramanian et al. An RNA aptamer directed against the primary miR-17-92 transcript was selected by the systemic evolution of ligands by exponential enrichment (SELEX) to inhibit the biogenesis of the miRNA cluster. The authors confirmed the inhibition of cluster biogenesis and a decrease in Rb cell proliferation (Subramanian et al., 2015).

However, the described investigations focused mainly on a single element (miRNA or gene). Li et al. studied genes, miRNAs, and transcription factors as elements of the regulatory networks, analyzing their relationship in Rb. The authors focused on the interactions between genes, which regulate miRNAs, and host genes including miRNAs and miRNAs targeting genes. The authors thus constructed three regulatory networks based on the relationships called the related network, differentially expressed network and global network. Data on Rb-related genes for the related network were obtained from GeneCards database and pertinent literature, whereas data on differently expressed genes in Rb for the differentially expressed network were obtained from the Cancer Genetics Web. Expressed miRNAs were extracted from literatures and mir2Disease, which is a database about differentially expressed miRNAs in various human diseases. Rb-related miRNAs were collected manually from permanent literatures.

The global network was constructed from all of the interactions that have been experimentally validated, making it too complex to be used or obtaining information. In this way, the authors were able to identify the pathways of the related elements and differentially expressed elements inserted in the other two networks (Li et al., 2014).

All of the above miRNAs are summarized in **Table 2**.

**Table 2:** microRNAs (miRNAs) involved in retinoblastoma (Rb) and some of their putative target genes.

miRNA	miRBase accession number/HGNC ID	Expression in Rb	Target (reference)
**miR-138-5p**	MIMAT0000430 –	Downregulated in RB cell lines	*PDK1*(Wang et al., 2017)
**miR-485**	MI0002469 HGNC:32067	Downregulated in RB tissues and cell lines	*Wnt3A* (Lyu et al., 2019b)
**miR-186**	MI0000483 HGNC:31557	Downregulated in RB cell lines	*DIXDC1* (Che et al., 2018)
**miR-504**	MI0003189 HGNC:32139	Downregulated in RB tissues and cell lines	*AEG-1* (Wang et al., 2019)
**miR-361-3p**	MIMAT0004682 –	Downregulated in RB tissues serum samples and cell lines	*GLI 1/3* (Zhao and Cui, 2018)
**miR-31**	MI0000089 HGNC:31630	Downregulated in RB cell lines	(Montoya et al., 2015)
**miR-200a**	MI0000737 HGNC:31578	Downregulated in RB cell lines	(Montoya et al., 2015)
**miR-183**	MI0000273 HGNC:31554	Downregulated in RB tissues and cell lines	*LRP6* (Wang et al., 2014)
**miR-98**	MI0000100 HGNC:31649	Downregulated in RB tissues and cell lines	*IGF1R* (Guo L, et al., 2019)
**miR-125a-5p**	MIMAT0000443 –	Downregulated in RB tissues and cell lines	*TAZ* (Zhang et al., 2016)
**miR-204**	MI0000284 HGNC:31582	Downregulated in RB tissues and cell lines	*CyclinD2, MMP-9* (Wu et al., 2015; Golabchi et al., 2017)
**miR-29a**	MI0000087 HGNC:31616	Downregulated in RB tissues and cell lines	*STAT3* (Liu et al., 2018)
**miR-125b-5p**	MIMAT0000423 –	Upregulated in RB tissues and cell lines	*DRAM2* (Bai et al., 2016)
**miR-492**	MI0003131 HGNC:32081	Upregulated in RB tissues and cell lines	*LATS2* (Sun et al., 2019)
**miR-24-3p**	MIMAT0000080 –	Upregulated in RB tissues and cell lines	*p14ARF* (To et al., 2012)
**miR-21**	MI0000077 HGNC:31586	Upregulated in RB cell lines	*PDCD4* (Shen et al., 2014)
**miR-106b**	MI0000734 HGNC:31495	Upregulated in RB cell lines	*Runx3* (Yang et al., 2017)

## lncRNAs Pathways in Rb

In the same way as miRNAs, lncRNAs are responsible for various cellular functions and play an important role in many cancer types as biomarkers, tumor regulators, and predictors of prognosis. Evidence has emerged of interesting pathways involving lncRNAs and their putative gene targets in different tumors. Musahl et al. demonstrated that the depletion of the ncRNA-RB1, an lncRNA expressed by the RB1 promoter, reduced the expression of calreticulin (CARL). CARL is an endoplasmatic reticulum protein that, in pre-apoptosis, translocates to the cell surface and serves as a signal to phagocytic cells. As a result of ncRNA-RB1 depletion, tumor cell uptake by macrophages is inhibited (Musahl et al., 2015). E2F1-regulated inhibitor of cell death (ERIC) is also capable of controlling DNA damage response and subsequent apoptotic cell death, interacting with proliferation regulators such as E2F1. Specifically, Feldstein et al. showed that ERIC is regulated at transcriptional level by E2F1 and responds to DNA damage in osteosarcoma and lung cancer cell lines. The authors observed an upregulation of ERIC after DNA damage that regulated apoptosis. Consequently, the inhibition of ERIC expression led to increased apoptosis (Feldstein et al., 2013).

lncRNAs are involved in Rb progression. Li et al. observed that lncRNA 00152 (LINC00152) was upregulated in Rb cell lines and tissues. The authors also reported that Rb cells silenced for LINC00152 showed an inhibition of cell proliferation, migration, invasion, and colony formation, and increased apoptosis. Moreover, LINC00152 knockdown caused the activation of caspase-3 and caspase-8 *in vitro* and suppressed tumorigenesis in nude mouse models (Li et al., 2018).

Dong et al. reported that HOTAIR lncRNA is involved in Rb progression through the Notch signalling pathway. HOTAIR is an oncogenic lncRNA correlated with metastasis, invasion, tumorigenesis and drug resistance. It is significantly upregulated in human Rb tissues and the authors showed that lncRNA knockout impeded the proliferation of Y-79 cell line. Notch also plays an important role in tumor development processes. In particular, Dong et al. assessed the expression levels of Notch1 and Jagged 1, the most common ligand and receptor, respectively, in the Notch pathway. They found that Jagged 1 and Notch 1 expression decreased after HOTAIR knockdown, indicating that HOTAIR regulates tumor progression in Rb through the activation of the *Notch 1* pathway (Dong et al., 2016). Another mechanism of action of HOTAIR is that of a miRNA sponge. Yang et al. demonstrated that HOTAIR sponged miR-613, which subsequently did not trigger tyrosine protein kinase met (c-met), its direct target gene. *C-met* is a protoncogene and its upregulation through HOTAIR causes the progression of Rb (Yang et al., 2018).

The testis-associated highly conserved oncogenic lncRNA (THOR) has been identified as another Rb promoter. THOR is widely expressed in various cancer types including Rb, but is also restrictively expressed in healthy testis tissues. Shang et al. showed that THOR promoted the malignant phenotype of Rb by interacting with insulin-like growth factor 2 mRNA binding protein 2 (IGF2BP1) and by controlling the mRNA stability of the c-myc oncogene. C-myc must be associated with IGF2BP1 to prevent its degradation. Thus in Rb cells, upregulated THOR promotes the association of c-myc and IGF2BP1, leading to its stabilization and consequently enhancing the malignant phenotype (Shang, 2018).

Wang et al. discovered that the lncRNA differentiation antagonizing non-protein coding RNA (DANCR) is upregulated in Rb cell lines and tissues and also overexpressed in Rb patients, leading to poor overall and disease-free survival. The authors observed that DANCR sponged miR-34c and miR-613, two miRNAs with tumor suppression function that target matrix metallopeptidase 9 (MMP9), an important protein for the breakdown of ECM. When DANCR regulation was activated, MMP9 was upregulated, leading to tumor progression (Wang et al., 2018).

A further connection between lncRNA and miRNA in Rb was demonstrated by Zhang et al. in their investigation of the association between lncRNA CCAT1 and miR-218-5p, known to occur in other cancer types. In fact, Lu et al. had previously shown that the negative regulation of miR-218-5p by CCAT1 promoted tumor progression in lung cancer (Lu et al., 2016). In the same way, Zhang et al. proved that CCAT1 was upregulated in Rb. Confirming the same mechanism of negative interaction with miR-218-5p, the authors showed that there was a reduction in apoptosis and an increase in cell proliferation and migration capacity (Zhang et al., 2017).

Actin filament-associated protein 1-antisense RNA 1 (AFAP1-AS1) is yet another lncRNA that has been hypothesized as having an oncogenic function in Rb. This lncRNA is associated with cancer progression and has been found to be overexpressed in various cell lines and tumor types such as lung cancer, ovarian cancer, oesophageal cancer, gastric cancer, hepatocellular carcinoma, nasopharyngeal carcinoma, colorectal cancer, biliary tract cancer, and pancreatic ductal adenocarcinoma (Zhang et al., 2018b). The role of this lncRNA in Rb was unknown up until recently. Hao et al. compared normal retina cell lines with Rb cells in knockdown experiments, observing that AFAP1-AS1 downregulation inhibited cell cycle progression, invasion, and migration. The authors also confirmed the oncogenic function of AFAP1-AS1 through its upregulation in Rb, reporting that it caused larger tumor size and optic nerve and choroidal invasion (Hao et al., 2018).

BDNF antisense RNA (BDNF-AS) is an lncRNAs transcribed by RNA polymerase II and has proved to be reverse regulator of BDNF. BDNF is a member of the neurotrophin family of growth factors whose role is to facilitate neuron survival and support the differentiation and growth of new synapses and neurons. Shang et al. evaluated the expression of BDNF-AS in Rb cell lines and found that it was downregulated in both Rb cell lines and tissues. Conversely, they also demonstrated that forced expression of BDNF-AS diminished cancer proliferation and metastatic potential, arresting cells in Go/G1 phase and consequently downregulating the cell cycle-associated proteins cyclin E and CDC42 (Shang et al., 2017).

Another lncRNA associated with tumorigenesis is promoter of CDKN1A antisense DNA damage activated RNA (PANDAR), and there is evidence of its upregulation in several types of cancers (Peng and Fan, 2015; Jiang et al., 2017). Sheng et al. investigated the potential clinical role of PANDAR in Rb, observing that it was overexpressed in Rb cells and tissues and that it inhibited cell apoptosis by affecting the *Bcl-2/caspase-3* pathway. Moreover, using online databases, the authors predicted the specificity protein 1 (SP1) as a potential transcriptional factor capable of binding directly to the PANDAR promoter region and of triggering its transcription. They subsequently confirmed *in vitro* this binding ability of SP1 to the PANDAR promoter region.

The new connection discovered between PANDAR and SP1 could represent an alternative therapeutic target for Rb (Sheng et al., 2018).

In addition to the other lncRNAs, BRAF-activated non-coding RNA (BANCR) also plays an important role in the progression of various cancers, including Rb. Su et al. demonstrated that BANCR was overexpressed in Rb cell lines and tissues and that it was associated with tumor development. In fact, knocked down BANCR limited tumor invasion, metastatic capacity, and proliferation, indicating its potential usefulness as a therapeutic target for Rb (Su et al., 2015).

lncRNA H19 was among the first lncRNAs to be discovered and has different functions in different cancers. Zhang et al. hypothesized that H19 may also be involved in Rb. Initially, the authors assessed H19 expression in Rb, observing that it was substantially downregulated in Rb tissues and cell lines. They also showed that H19 has seven binding sites where it directly binds the miR-17-92 cluster in a competitive way. It has been already seen that the miR-17-92 cluster suppresses p21, an important regulator of the cell cycle, leading to STAT3 activation. Zhang et al. showed that H19 inhibited the suppressor role of the miR-17-92 cluster in p21 gene, decreasing STAT3 activation (Zhang et al., 2018a).

Cheng et al. observed that lncRNAs X inactive specific transcript (XIST) had an oncogene function in Rb. The authors discovered that XIST binds miR-101, regulating the repression of its targets, E-box binding homeobox 1 and 2 (ZEB1, ZEB2). ZEB1 and ZEB2 are transcription factors that are responsible for the malignant features of different type of cancers. The binding of miR-101 to XIST and the subsequent oncogenic activity of ZEB1 and ZEB2 led to increased proliferation, invasion and migration of Rb cells (Cheng et al., 2019).

A similar mechanism was uncovered by Liu et al. who found that the lncRNA metastasis-associated lung adenocarcinoma transcript 1 (MALAT1) was upregulated in Rb. The authors observed that MALAT1 silencing in cells caused the inhibition of cell viability, invasion and migration, activated apoptosis and upregulated miR-124.

They also demonstrated that MALAT1 sponged miR-124 which, consequently, did not target E-cadherin transcription repressor (Slug), its target gene. Slug activates ERK/MAPK and Wnt/β-catenin pathways and, when downregulated by miR-124, inhibits Rb progression (Liu S, et al., 2017).

The above-mentioned lncRNAs are summarized in **Table 3**.

**Table 3:** Long ncRNAs (lncRNAs) involved in retinoblastoma (Rb) and some of their putative target genes.

lncRNA	HGNC ID	Expression in Rb	Target (reference)
**H19**	HGNC:4713	Downregulated in RB tissues and cell lines	*miR-17-92 cluster* (Zhang et al., 2018a)
**BDNF-AS**	HGNC:20608	Downregulated in RB tissues and cell lines	(Shang et al., 2017)
**hsa_circ_0001649**	–	Downregulated in RB tissues and cell lines	*AKT/mTOR pathway* (Xing et al., 2018)
**TET1hsa_circ_0093996**	–	Downregulated in RB tissues	*miR-183* (Lyu et al., 2019a)
**HOTAIR**	HGNC:33510	Upregulated in RB tissues and cell lines	*Notch* (Dong et al., 2016) *miR-613* (Yang et al., 2018)
**MALAT1**	HGNC:29665	Upregulated in RB cell lines	*miR-124* (Liu S, et al., 2017)
**DANCR**	HGNC:28964	Upregulated in RB tissues and cell lines	*miR-34c, miR-613* (Wang et al., 2018)
**AFAP1-AS1**	HGNC:28141	Upregulated in RB tissues and cell lines	(Hao et al, 2018)
**BANCR**	HGNC:43877	Upregulated in RB tissues and cell lines	(Su et al., 2015)
**PANDAR**	HGNC:44048	Upregulated in RB tissues and cell lines	*Bcl2/caspase 3 pathway* (Sheng et al., 2018)
**LINC00152**	HGNC:28717	Upregulated in RB tissues and cell lines	*caspase 3 and caspase 8* (Li et al., 2018)
**THOR**	HGNC:53788	Upregulated in RB tissues and cell lines	*c-myc* (Shang, 2018)
**CCAT1**	HGNC:45128	Upregulated in RB tissues and cell lines	*miR-218-5p* (Zhang et al., 2017)
**XIST**	HGNC:12810	Upregulated in RB tissues and cell lines	*miR-101* (Cheng et al., 2019)

## 

## Circular RNAs Pathways in Rb

Circular RNAs (circ-RNAs) are an emerging group of ncRNAs present inside cells. Considering their form, they are less susceptible to RNAs degradation and are the result of a back-splicing of 5’splice position with the 3’splice position or exon skipping mechanism. Recent evidence suggests that various disorders such as nervous diseases, vascular inflammation and several types of cancer may be the result of their deregulation (Guo N, et al., 2019).

hsa_circ_0001649 is a new cancer associated circ-RNA and is reported to be the transcription product of Snf2 Histone Linker Phd Ring Helicase (SHPRH) tumor suppressor gene. Xing et al. showed *via* q-RT PCR analysis that hsa_circ_0001649 was downregulated in Rb tissue samples compared with normal tissue and that low expression of this circ-RNA was associated with aggressive phenotypes in Rb patients. To further confirm its involvement in tumorigenesis, the authors assessed hsa_circ_0001649 enhanced expression in xenografts, reporting that tumors formed from hsa_circ_0001649-transfected cells were smaller than those of the control group. In the same study, the authors investigated the molecular mechanisms of hsa_circ_0001649 which are responsible for changes in Rb tumor cell proliferation. The Akt/mTOR apoptosis-related signalling pathway is known to be related to Rb progression, but there is still no evidence of the involvement of circ-RNA. Xing et al. suggested that the Akt/mTOR signalling pathway is regulated by hsa_circ_0001649. More precisely, after transfection of Rb cell lines with hsa_circ_0001649, the authors observed that p-AKT and p-mTOR were negatively correlated with hsa_circ_0001649 (Xing et al., 2018).

In another study, Lyu et al. analyzed the expression profile of circ-RNA in human Rb tissue and in corresponding normal retina, observing a general reduction in circ-RNA expression levels in Rb. This may have been a result of compromised back-splice machinery in the circ-RNA production or by an excessive consumption of circ-RNA, necessary for cell proliferation. In particular, TET1-hsa_circ_0093996 was significantly downregulated in Rb tissue. The authors also observed a downregulation of tumor suppressor PDCD4. Based on the *in silico* analysis, which revealed that miR-183 targeted PDCD4, the authors hypothesized that TET1-hsa_circ_0093996 sponged miR-183. In an attempt to create a regulatory axis, they assumed that TET1-hsa_circ_0093996 downregulation increased unbound miR-183, which consequently targeted PDCD4 causing enhanced cell proliferation (Lyu et al., 2019a).

## Conclusions

ncRNAs are molecules physiologically present in humans where they regulate gene expression after transcription and subsequently control important mechanisms such as cell proliferation, development, and apoptosis. ncRNAs are deregulated in many types of cancer, suggesting that they are involved in carcinogenesis. In the present review, we explored short and long ncRNAs, which are deregulated in Rb, demonstrating that, in addition to *RB1,* there is a great number of other molecules involved in the development of the malignant phenotype of this type of cancer.

The evolution of genomic and genetic technologies together with the generation and development of bioinformatics have made it possible to manage the enormous quantity of accumulated data generated by large-scale high throughput analyses and basic research.

Profiling analysis is the first step to discover new mechanisms of ncRNAs involved in Rb development.

miRNA profiles in Rb have been discussed at length in this review (Beta et al., 2013; Yang and Mei, 2015). The studies in question identified miRNAs that are upregulated and downregulated in Rb with an oncogene or tumor suppressor function, respectively.

The function of miRNAs is explicated through the regulation of target genes involved in cell proliferation, migration, invasion, cell viability, and apoptosis, as demonstrated by Wang et al. (2017). Through a comparison of miRNAs and gene profiling it is possible to identify the pathways that determine Rb progression. These pathways are carefully validated through *in vitro* and *in vivo* experiments. miRNAs directly or indirectly regulate important gene such as cyclins (Wu et al., 2015; Golabchi et al., 2017) and TP53 (To et al., 2012; Bai et al., 2016), respectively. In turn, lncRNAs regulate miRNAs through sponge mechanism (Liu S, et al., 2017; Wang et al., 2018; Yang et al., 2018; Zhang et al., 2018a). Studies show the complex ncRNA mechanisms and the prevailing pathways that determine Rb progression.

Further research into the complex Rb pathways will help to identify novel ncRNA-based therapeutic approaches to counteract the aberrations of the ncRNA that are responsible for the development of Rb. Several studies described in this review analyzed the therapeutic potential of ncRNAs, but although they highlighted the therapeutic prospects of these molecules, their clinical implementation remains a challenge. A non-toxic delivery system is needed to selectively transport ncRNA-based therapeutics to the tumor site, e.g., antisense oligonucleotide or inhibitor against an oncogene (Adams et al., 2017). Moreover, the fact that a single miRNA binds more than 100 target genes makes target specificity a problem. The recent discovery that ncRNAs contained in exosomes interact with the tumor microenvironment and affect cancer growth and metastatic potential has opened up a new chapter on the intercellular crosstalk for cancer biology (Vannini et al., 2018a). Thus, new strategies to impair the exosome-mediated ncRNA transfer affecting cancer growth and dissemination can be hypothesized. The characterization of regulatory mechanisms of lncRNAs is also a critical aspect to complement the deficiency of precision medicine.

A better understanding of the role played by ncRNAs in chemoresistance would enable patients to be spared from non-beneficial treatments but would also help to overcome the problem by modulating the expression of the ncRNAs involved in the resistance mechanisms.

Future studies will serve to clarify the role of ncRNAs as tumor suppressors or oncogenes and to design new ncRNA-based therapeutic approaches. The era of an ncRNA-based therapy for rb is fast approaching and will provide oncologists with a powerful tool for improving patients’ odds against this often deadly tumor.

## Author Contributions

MP found the articles that describe ncRNAs in Retinoblastoma and she wrote the manuscript. IV wrote and corrected the manuscript.

## Conflict of Interest

The authors declare that the research was conducted in the absence of any commercial or financial relationships that could be construed as a potential conflict of interest.
